# Imaging evaluation of the cartilage in rheumatoid arthritis patients with an x-ray phase imaging apparatus based on Talbot-Lau interferometry

**DOI:** 10.1038/s41598-020-63155-9

**Published:** 2020-04-16

**Authors:** Hiroyuki Yoshioka, Yuho Kadono, Yoon Taek Kim, Hiromi Oda, Takashi Maruyama, Yuji Akiyama, Toshihide Mimura, Junji Tanaka, Mamoru Niitsu, Yoshihide Hoshino, Junko Kiyohara, Satoshi Nishino, Chiho Makifuchi, Atsushi Takahashi, Yuko Shinden, Norihiro Matsusaka, Kazuhiro Kido, Atsushi Momose

**Affiliations:** 1 Department of Orthopaedic Surgery, Saitama Medical University, Moroyama, Japan; 2 Department of Rheumatology and Applied Immunology, Saitama Medical University, Moroyama, Japan; 3 Department of Radiology, Saitama Medical University, Moroyama, Japan; 4 Corporate R&D Headquarters, Konica Minolta, Inc., Hachioji, Japan; 5 Institute of Multidisciplinary Research for Advanced Materials, Tohoku University, Sendai, Japan

**Keywords:** Preclinical research, Rheumatoid arthritis

## Abstract

X-ray Talbot-Lau interferometry is one of the x-ray phase imaging methods that has high sensitivity in depicting soft tissues. Unlike earlier x-ray phase imaging methods that required particular types of x-ray sources, such as a synchrotron or a micro-focus x-ray tube, x-ray Talbot-Lau interferometry enables to perform clinical x-ray phase imaging using a conventional x-ray source with a relatively compact configuration. We developed an apparatus to depict cartilage in the metacarpophalangeal joints of the hands. In addition, we examined the apparatus performance by applying it to healthy volunteers and patients with rheumatoid arthritis (RA). Cartilage deformation, which is thought to be a precursor of destruction of the joints, was successfully depicted by the apparatus, suggesting a potential early diagnosis of RA.

## Introduction

X-ray phase imaging is a promising method for depicting soft tissues because of its high sensitivity to materials consisting of light elements^1^. However, its clinical application has not yet been reported because it requires a coherent x-ray source, such as a huge synchrotron radiation source. X-ray grating interferometry or x-ray Talbot interferometry was demonstrated by Momose *et al*. for x-ray phase imaging^2^, making a breakthrough in this problem because a micro-focus x-ray source became available. However, the power of micro-focus X-ray sources is insufficient for clinical use. Talbot-Lau interferometry, which is a modification of Talbot interferometry, was first suggested by Clauser *et al*.^3^ and constructed for x-rays by Pfeiffer *et al*.^4^. This opened up the possibility for the development of clinical imaging apparatuses, which can be operated in a normal x-ray room with a conventional x-ray tube. X-ray phase imaging method acquires phase-contrast pictures by a digital image sensor and produces quantitative images after computer image processing^2,4^.

In the medical field, some animal and *ex vivo* human studies focused on depicting breast cancer^5^, lung diseases^6^, and brain diseases^7^. We, as well as Muehleman *et al*. and Stutman *et al*.^8,9^, have focused on depicting the limbs cartilage. After confirming the depiction on a cadaver and healthy volunteers^10,11^, we started the first clinical study on depicting the cartilage in patients with arthritis. We aimed to early diagnose rheumatoid arthritis (RA). Thanks to biological agents, such as anti-tumour necrosis factor (TNF)-alpha biologics and non-TNF biologics, which have evolved recently following disease-modifying antirheumatic drugs^12,13^, an early diagnosis, expected by the phase-imaging apparatus and successful treatments with biological agents, would be effective in suppressing progression of joint destruction.

In the present study, the apparatus based on Talbot-Lau interferometry^11^ was used to measure cartilage in the metacarpophalangeal (MP) joints of healthy volunteers and patients with RA. We also employed a magnetic resonance imaging (MRI) scanner for comparison. This study aimed to determine the benefit of using the apparatus to evaluate patients with RA.

## Results

### Images of healthy volunteers and RA patients

**Figure 1:**
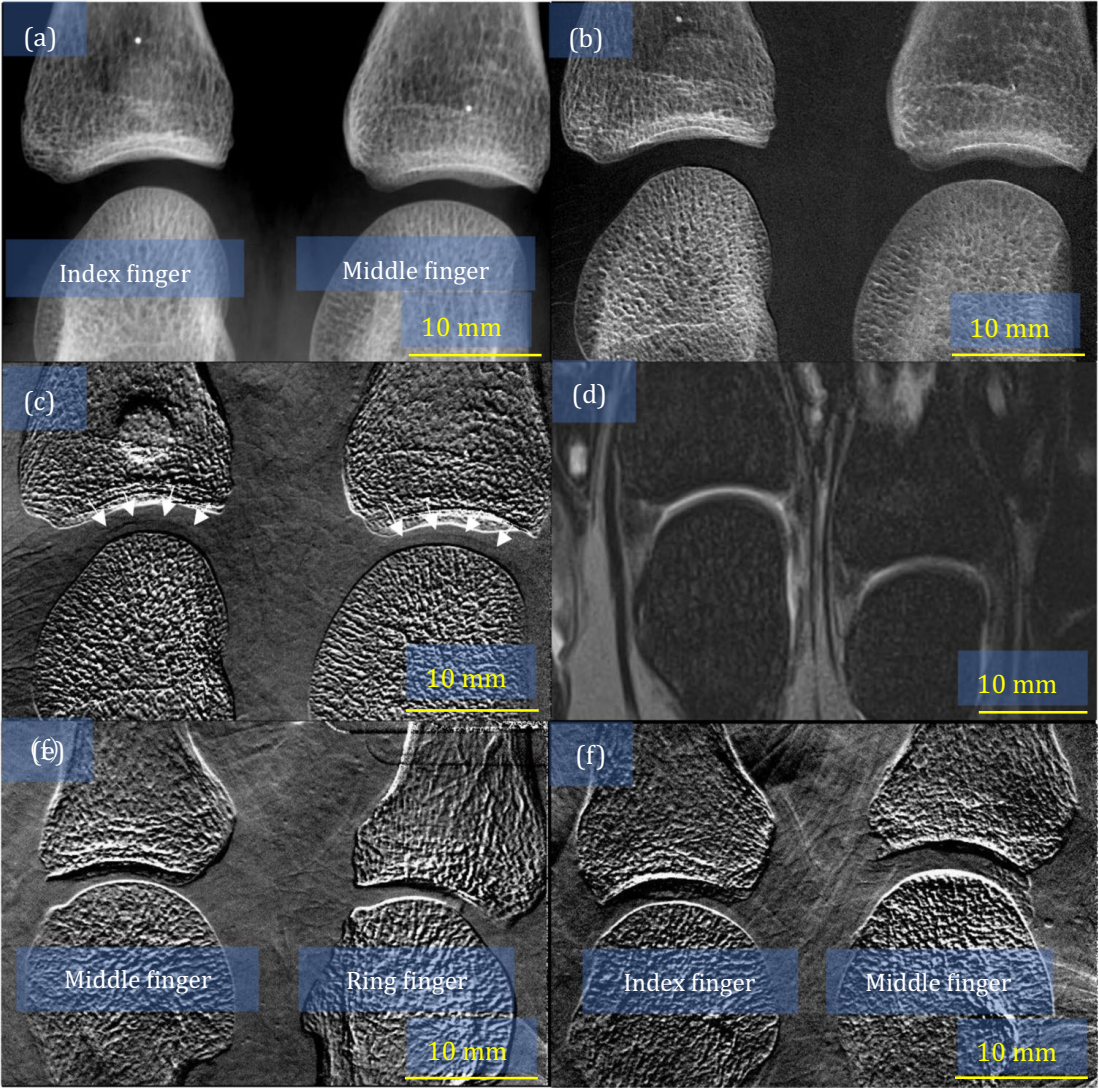
Images of metacarpophalangeal joints obtained from a healthy volunteer: (**a**) attenuation image, (**b**) small-angle-scattering image, (**c**) refraction image, where the arrows indicate the surface of the cartilage, and (**d**) magnetic resonance imaging scan. Images of the metacarpophalangeal joints that were not depicted properly: (**e**) an example of insufficient finger stretching in both fingers and (**f**) an example of the motion artefact in the middle finger.

We examined 2 MP joints selected from the index, middle, or ring finger per subject (140 joints in 70 patients, 110 joints in 55 healthy volunteers). The summary of depiction and the demographic background of the participants are shown in Table 1. Images of the left index and middle fingers of a healthy volunteer are shown in Fig. 1: (a) attenuation image, (b) small-angle-scattering image, (c) refraction image, and (d) MRI scan. The cartilage of the distal metacarpal is depicted in the refraction image, which is confirmed by comparing it with the MRI image. This result was consistent with those obtained from a cadaver and a healthy volunteer, reported previously^11^.

**Table 1:** Summary of the depiction and background of theparticipants.

	Number of joints (subjects)	Averaged ages (years)	Men:Women	Larsen grade (0/I/II/III/IV/V)	Number of measurable joints	Larsen grade (measurable) (0/I/II/III/IV/V)
Healthy volunteers	110 (55)	43 (23–66)	15:40	—	37	—
RA patients	140 (70)	60 (26–80)	12:58	106/22/6/2/4/0	42	30/11/1/0/0

The cartilage in 79 joints was properly depicted and measured, and we were unable to measure the cartilage in other joints. One reason for this was that the cartilage was obstructed by the metacarpal bone due to insufficient finger stretch by the holder (133 joints) Fig. 1(e). Another reason was the artefact caused by the patients movement during the exposure (17 joints) (Fig. 1(f)).

**Figure 2:**
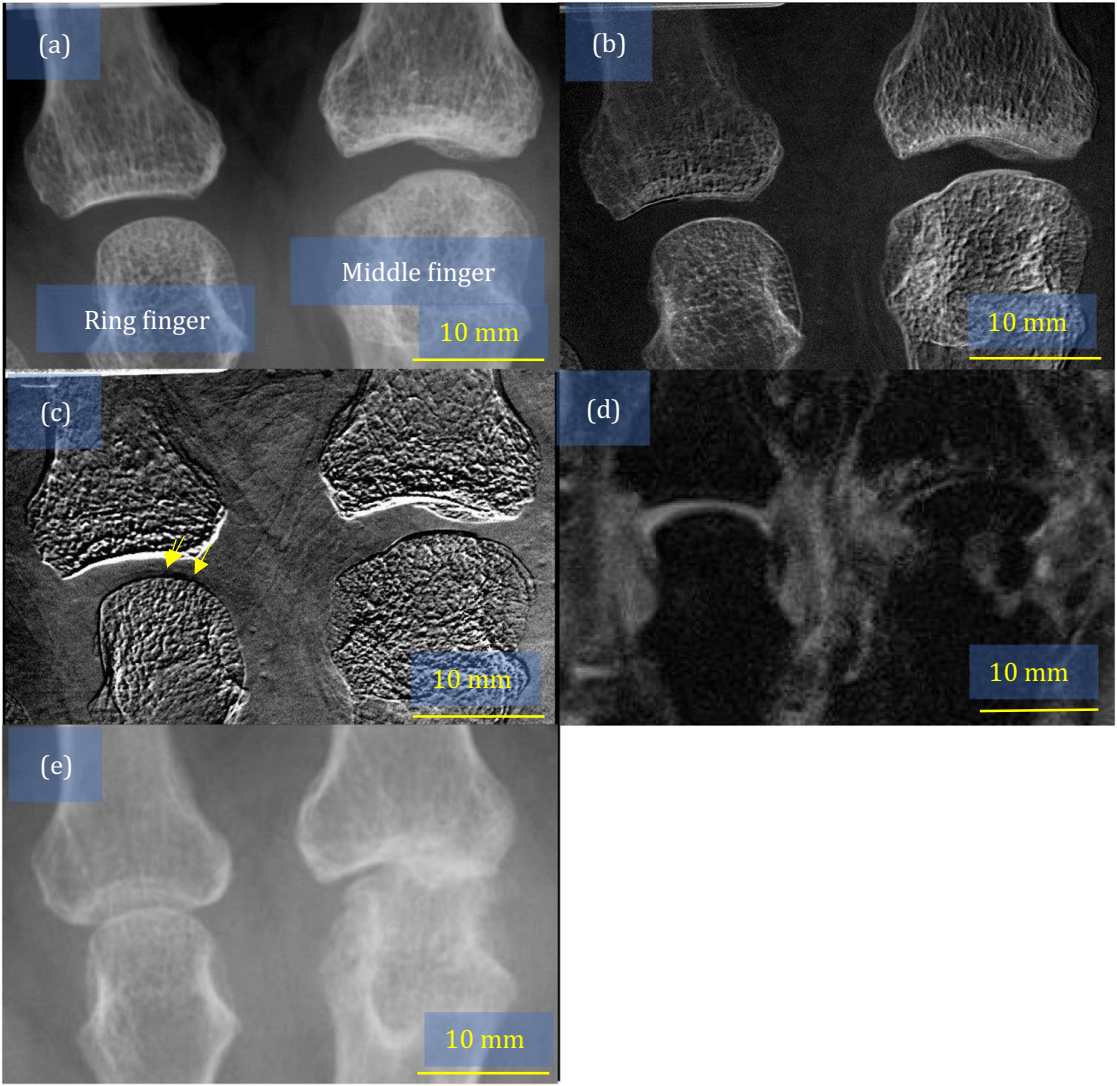
Images of metacarpophalangeal joints in the middle and ring fingers obtained from patient A: (**a**) attenuation image, (**b**) small-angle-scattering image, (**c**) refraction image where the yellow arrows indicate the partial thinning of the cartilage, (**d**) magnetic resonance imaging scan, and (**e**) conventional x-ray image, which was used for evaluating the Larsen grade.

**Figure 3:**
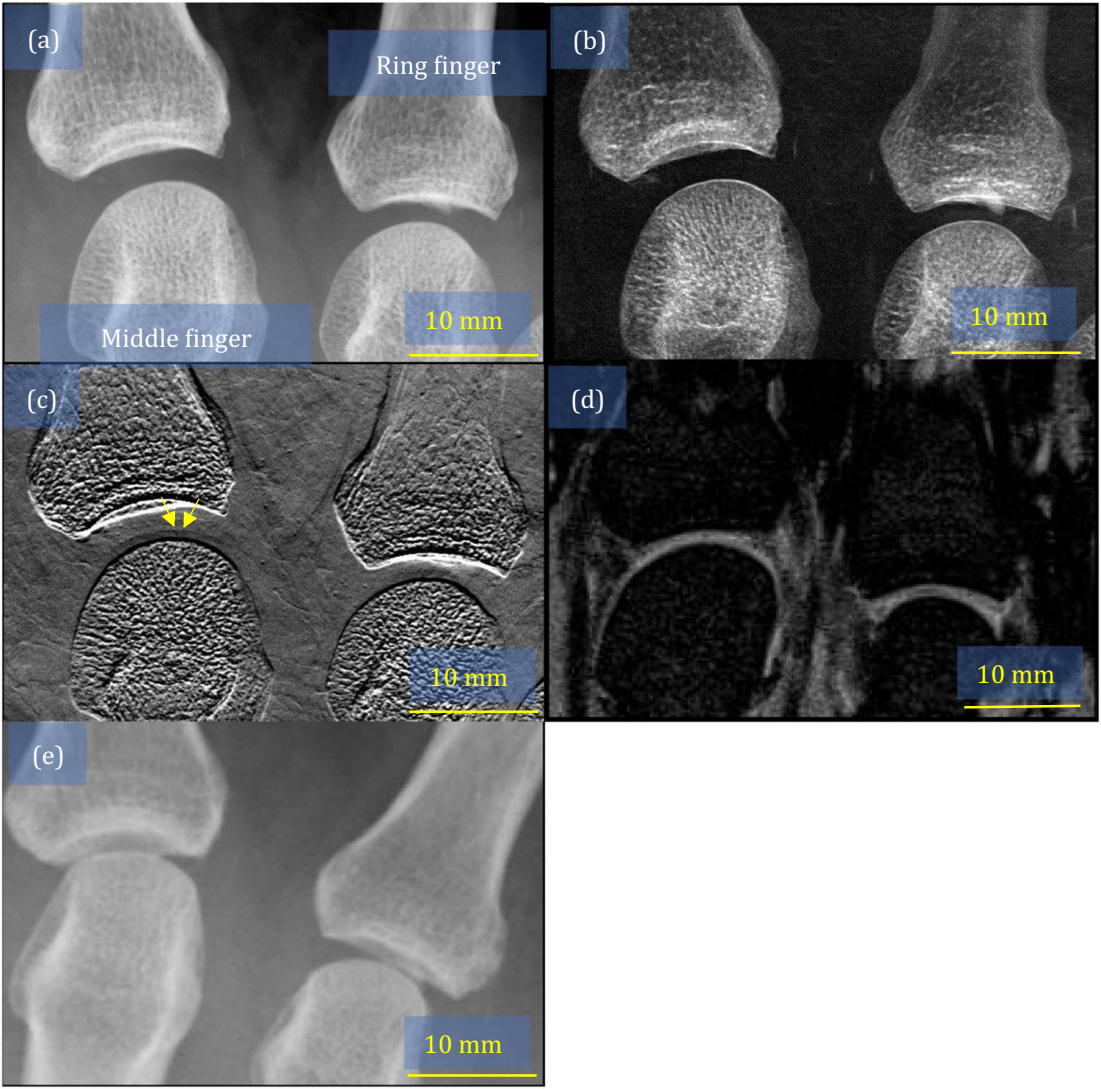
Images of metacarpophalangeal joints in the middle and ring fingers obtained from patient A: (**a**) attenuation image, (**b**) small-angle-scattering image, (**c**) refraction image, where the yellow arrows indicate the partial thinning of the cartilage, (**d**) magnetic resonance imaging scan, and (**e**) conventional x-ray image, which was used for evaluating the Larsen grade.

The background, of the representative patients with RA named patients A and B, is shown in Table 2. The patients A and B images are shown in Figs. 2(a–e) and 3(a–e), respectively. In Figs. 2(e) and 3(e), conventional x-ray images that were used to evaluate Larsen grade are shown. We found that the cartilage was thin in the ring finger of patient A, as the yellow arrows in Fig. 2(c) indicate. Additionally, we observed partial cartilage deformation in the middle finger of patient B, as indicated by the yellow arrows in Fig. 3(c). The ring finger of patient B seemed obscured in a comparison with the images of a healthy volunteer in Fig. 1(c). MRI images that are shown in Figs. 2(d) and 3(d) also depict the cartilage thinning in the ring finger of patient A, and middle and ring fingers of patient B, suggesting that the findings by our apparatus were consistent with those of the MRI.

**Table 2:** Background characteristics of the representative patients with RA.

Patient ID	Sex	Age (years)	Duration of RA (years)	RF	MMP-3	Anti-CCP antibody	DAS28 (E/C)	CRP	Drug	Steinbrocker Stage	Target	Larsen Grade
A	Female	61	10	28.6	34.3	7	2.30	2.09	Etanercept	III	Right hand, middle finger	3
											Right hand, ring finger	2
B	Female	56	12	54.0	52.6	4.7	5.90	—	Methotrexate	II	Left hand, middle finger	1
											Left hand, ring finger	2

RA, rheumatoid arthritis; CRP, C-reactive protein; MMP-3, matrix metalloproteinase-3; CCP, cyclic citrullinated peptide; DAS-28, Disease Activity Score-28; RF, rheumatoid factor.

### Thickness of the cartilage

**Figure 4:**
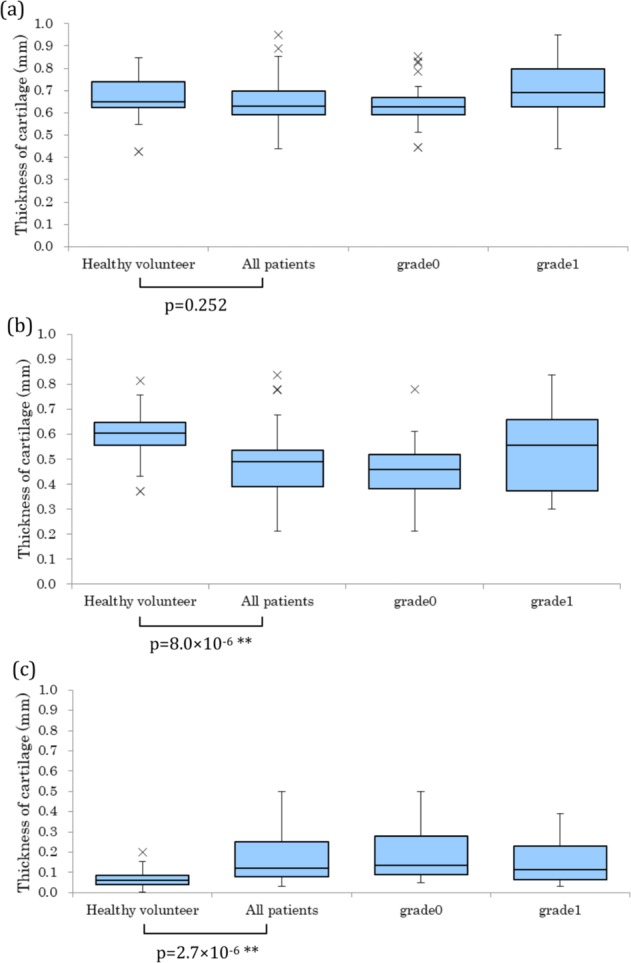
Measured thicknesses of the cartilage: (**a**) thickness of the cartilage in the whole area, (**b**) thickness in the minimum area, and (**c**) difference between (**a**) and (**b**).

Cartilage thickness, measured by the procedure, is described in the Methods section and shown in Fig. 4(a–c). A significant difference was found in the cartilage thickness between the healthy volunteers and patients with RA; the difference between the minimum thickness (minimum area) and average thickness (whole area) is remarkable. Since 98% of the measured fingers of patients were scored as grade 0 or grade I by Larsen Grade, the difference suggests that the apparatus could properly detect early deformation of the joints in RA patients.

## Discussion

Destroyed joints of patients with RA are generally irreparable. It was thought that joint destruction became remarkable within 5 years after the first appearance of symptoms^14–16^. Recently, it has been reported that destruction starts at 3 months after the first appearance of symptoms, and there is a possibility of preventing destruction with the use of methotrexate and biological agents at an early stage^12,17^. Therefore, it is important to detect the destruction as early as possible to diagnose RA and prevent deterioration of patients’ quality of life^18^. Seemingly, our apparatus is capable of detecting cartilage deformation as a precursor of joint destruction and expected to contribute to early determination of necessary medications required for proper treatment.

In general, conventional x-ray is used to evaluate RA, and the use of ultrasonic diagnostic equipment and MRI has been studied for evaluating RA^19,20^. Ultrasonic equipment is useful in diagnosing precise disease activity and remission due to its excellent sensitivity to synovitis. However, there are still some difficulties in introducing the equipment into diagnostic routines for outpatients because it has a longer inspection time than that by conventional methods, such as conventional x-ray. Furthermore, it requires skills for obtaining stable results. MRI has advantages in diagnosing RA in early stages, but it is also difficult to utilise the technology routinely because of long scan times and high initial costs for installation^21^.

We consider that our presented apparatus has a superior capability for early RA diagnosis. In addition, it is applicable to the daily clinic setting if improvements are made to the apparatus; that is, shortening the exposure time, expanding the field of view (FOV), and increasing the tube voltage to image thicker body parts other than the fingers.

In this paper, a preliminary summary of a clinical study was presented with more than 100 healthy volunteers and patients with RA. Significance of the x-ray phase-imaging apparatus has been demonstrated. Following this, detailed examination for early RA diagnosis will be continued by classifying imaging results by patients’ age, sex, and features.

## Methods

### Ethical statements

This study was approved by the institutional review ethics board of Saitama Medical University in Japan (application number: 692), and a written informed consent was obtained from each participant. The study was conducted in accordance with relevant guidelines and regulations.

### Imaging device

**Figure 5:**
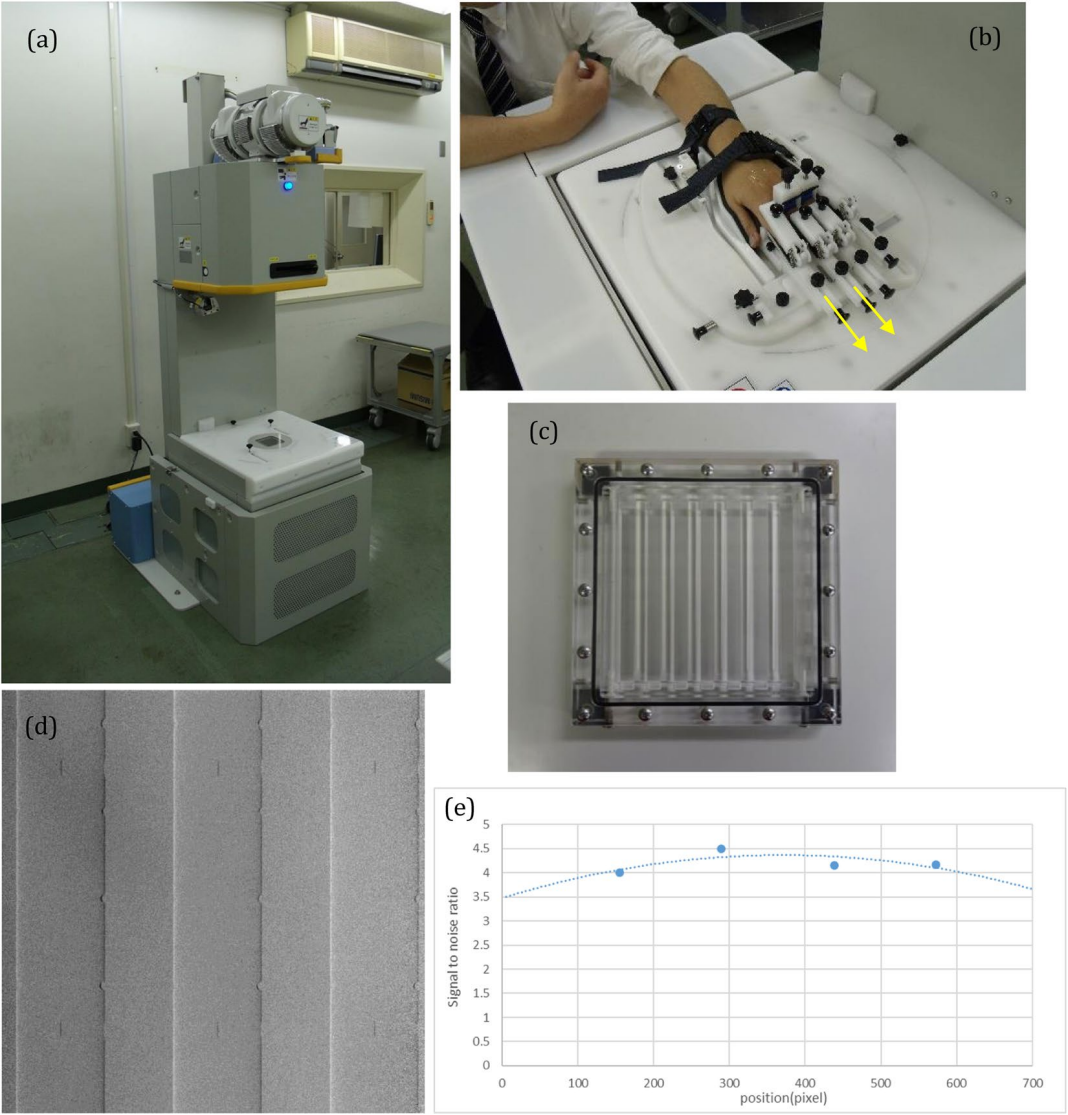
Imaging device: (**a**) whole view, (**b**) the holder for metacarpophalangeal joint imaging, with which the subject’s fingers are held and stretched to enlarge the joint spaces moderately in the direction shown by the yellow arrows, (**c**) image of a phantom consisting of polymethyl methacrylate pillars measuring 10 mm in diameter immersed in a water solution of dipotassium hydrogen phosphate (K_2_HPO_4_), (**d**) differential phase images of the phantom, and (**e**) signal-to-noise ratio at 4 mGy, at which the signal is defined as the averaged signal by refraction between the column and the water solution in 50 pixels and noise is defined as the standard division of the signal of the water solution in 50 × 50 pixels.

We used the apparatus for clinical use of Talbot-Lau interferometry^11^, of which the fundamental design was based on the simulation by Yashiro *et al*.^22^. The apparatus is shown in Fig. 5(a), which comprised an x-ray tube, a flat panel detector (FPD), a source grating (G0), a phase grating (G1), and an amplitude grating (G2). In addition, the FPD and the gratings are set in the housing of the apparatus. The x-ray tube (UH-6QC-307E; Hitachi Medical Corporation) had a tungsten anode and a nominal focal spot of 400 µm. The tube located above G0 was operated at 40 kVp and emitted x-rays downward. The x-ray mean photon energy was 28 keV. The specifications of the linear gratings mounted on the apparatus are shown in Table 3. The distance between G0 and G1 was 1.1 m, and that between G0 and G2 was 1.36 m. The FPD (Anrad, LMAM) was located beneath G2, and its pixel size was 85 µm. A target was placed 60 mm above G1, and the FOV was 46 mm × 46 mm, taking into account the magnification of the image when depicting the MP joints^11^. The values of modulation transfer function of the apparatus were 0.96 at 1 line/mm and 0.86 at 2 lines/mm in the width, and 0.92 at 1 line/mm and 0.73 at 2 lines/mm in the length at the position of the target. The apparatus was totally confirmed to satisfy the safety standards as a conventional x-ray system.

**Table 3:** Specifications of the linear gratings mounted on the apparatus.

	Pitch (μm)	Opening Width (μm)	Height (μm)
G0	22.8	7.00	110
G1	4.3	2.15	18
G2	5.3	2.65	100

G0, source grating; G1, phase grating; G2, amplitude grating.

The apparatus generated three kinds of images by the fringe-scanning method^1^; an attenuation image, a differential phase (or refraction) image, and a small-angle-scattering (or dark-field) image^23^. In this study, refraction images, which map the degree of x-ray deflection caused by the refraction in subjects, were evaluated in terms of the sensitivity to cartilage tissue. The period for the imaging sequence was 32 seconds, including 19 seconds of total x-ray exposure.

We focused on capturing images of the cartilage in MP joints of the fingers, which were the predilection sites of RA. We designed a holder to hold the participant’s wrist and each proximal phalanx so that the MP joints were positioned at a proper orientation for imaging. The holder, shown in Fig. 5(b), is equipped with a mechanism for adjusting the joint space that moderately stretched the subject’s fingers.

We also designed a phantom made of polymethyl methacrylate (PMMA) and a water solution (10.5% water) of dipotassium hydrogen phosphate (K_2_HPO_4_) so that the refractive-index difference between them was the same as that between the cartilage and joint fluid^24^. The phantom had round columns of PMMA measuring 10 mm in diameter; it was immersed in the water solution, and the thickness of the phantom was determined to be equivalent with the attenuation of x-rays through the finger joints, as shown in Fig. 5(c). We acquired images of the phantom at the same height as that for MP joint measurements and determined the dose as 4 mGy for human subjects to satisfy a signal-to-noise ratio larger than 3.0. The signal was defined as the averaged signal by refraction between the column and water solution in 50 pixels, and the noise was defined as standard division of the signal of the water solution in 50 × 50 pixels. The refraction images of the phantom with a 4 mGy dose and the signal to noise ratio are shown in Fig. 5(d,e). Peripheral areas showed high noise because of the reduction of the x-ray caused by gratings made on flat substrates.

### Evaluation of the cartilage

**Figure 6:**
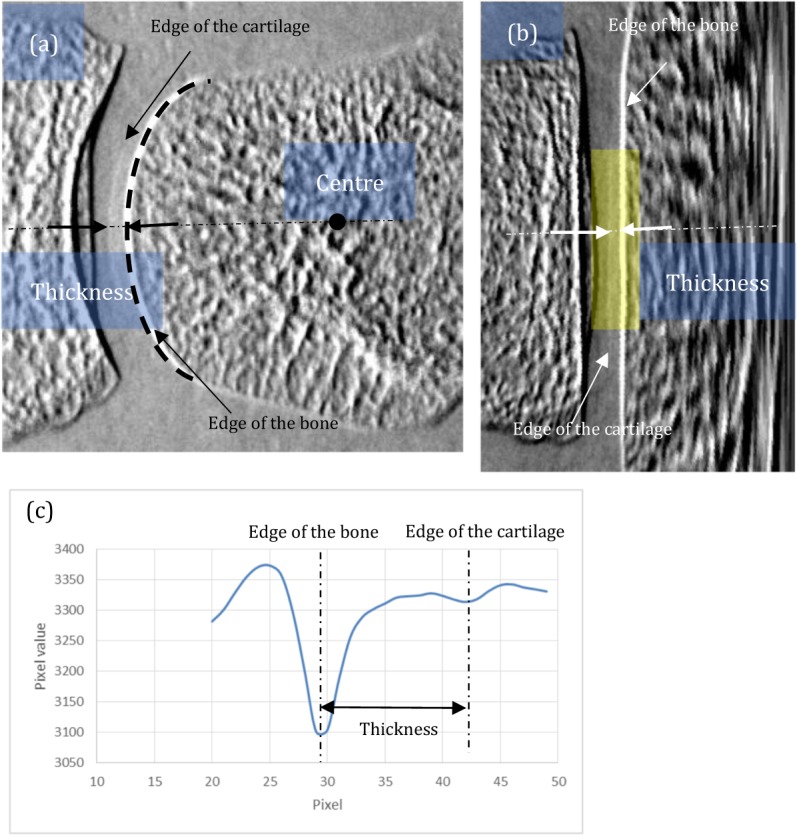
Method of measuring the cartilage: **(a**) the original image, with which the centre of curvature of the proximal phalanx was detected, (**b**) converted image so that the cartilage surface is almost flat, and (**c**) profile averaged over the yellow region in (**b**). Distance between the peaks corresponding to the surfaces of bone and cartilage is evaluated as the cartilage thickness.

We performed *in vivo* imaging of MP joints in 55 healthy volunteers and 70 patients with RA. We evaluated the condition of the cartilage in all fingers by measuring the thickness in the whole area and minimum area. The thickness in the whole area implies that the value averaged 17.8 mm along the cartilage surface in a refraction image. The thickness in the minimum area was the average value of 0.36 mm along the thinnest cartilage surface. The procedure of the thickness evaluation is shown in Fig. 6. First, the centre of curvature of the cartilage surface, depicted in a refraction image, was found (Fig. 6(a)). Then, the image was converted so that the surface of the cartilage was almost flat (Fig. 6(b)). The thickness was evaluated by measuring the distance between the peaks appearing in the averaged profile (Fig. 6(c)). The peaks were attributed to the surfaces of cartilage and bone. The range for profile averaging was 17.8 mm for the whole area thickness, as indicated by the yellow rectangle in Fig. 6(b). The joints of patients with RA were also evaluated by the Larsen grade with conventional x-rays^24^. Twenty-one patients were also examined by a 3-Tesla whole-body magnetic resonance scanner (SIEMENS, Skyra). For finger MRI, fat-saturated proton density weighted images with a time of repetition/time of echo of 2500/16 ms, FOV of 80 mm, and section thickness of 1.3 mm were obtained.

## Data Availability

The datasets generated during and/or analysed during the current study are available from the corresponding author on reasonable request.
